# Essential oils from *Dysphania* genus: Traditional uses, chemical composition, toxicology, and health benefits

**DOI:** 10.3389/fphar.2022.1024274

**Published:** 2022-12-08

**Authors:** Amal Dagni, Simona Codruta Hegheș, Ramona Suharoschi, Oana Lelia Pop, Adriana Fodor, Romana Vulturar, Angela Cozma, Oufaa Aniq filali, Dan Cristian Vodnar, Abdelaziz Soukri, Bouchra El Khalfi

**Affiliations:** ^1^ Laboratory of Physiopathology, Molecular Genetics and Biotechnology, Faculty of Sciences Ain Chock, Health and Biotechnology Research Centre, Hassan II University of Casablanca, Casablanca, Morocco; ^2^ Department of Drug Analysis, “Iuliu Hațieganu” University of Medicine and Pharmacy, Cluj-Napoca, Romania; ^3^ Department of Food Science, University of Agricultural Science and Veterinary Medicine of Cluj-Napoca, Cluj-Napoca, Romania; ^4^ Molecular Nutrition and Proteomics Lab, CDS3, Life Science Institute, University of Agricultural Science and Veterinary Medicine of Cluj-Napoca, Cluj-Napoca, Romania; ^5^ Clinical Center of Diabetes, Nutrition and Metabolic Diseases, “Iuliu Haţieganu” University of Medicine and Pharmacy, Cluj-Napoca, Romania; ^6^ Department of Molecular Sciences, “Iuliu Hațieganu” University of Medicine and Pharmacy, Cluj-Napoca, Romania; ^7^ Cognitive Neuroscience Laboratory, Department of Psychology, Babeș-Bolyai University, Cluj-Napoca, Romania; ^8^ Internal Medicine Department, 4th Medical Clinic “Iuliu Haţieganu” University of Medicine and Pharmacy, Cluj-Napoca, Romania; ^9^ Food Biotechnology and Molecular Gastronomy, CDS7, Life Science Institute, University of Agricultural Science and Veterinary Medicine of Cluj-Napoca, Cluj-Napoca, Romania

**Keywords:** *Dysphania*, ethnophamacology, essential oils, medicinal benefits, toxicology

## Abstract

The genus *Dysphania* belongs to the Amaranthaceae family and is known for its many health benefits. Therefore, it is commonly available worldwide and includes more than 47 species, five species have been mainly reported, and *D. ambrosioides* has been one of the most widely used plants for thousands of years as a remedy for a wide range of ailments. In recent investigations, the essential oils of the genus *Dysphania* have been examined for their antibacterial, antioxidant, and antiviral properties related to specific components such as terpenoid compounds that exhibit pharmacological activity. Moreover, some of *Dysphania*’s compounds show a toxicological effect. Therefore, the objective of the study was to provide EO chemical composition and pharmacological data of the genus *Dysphania*.

## Graphical Abstract

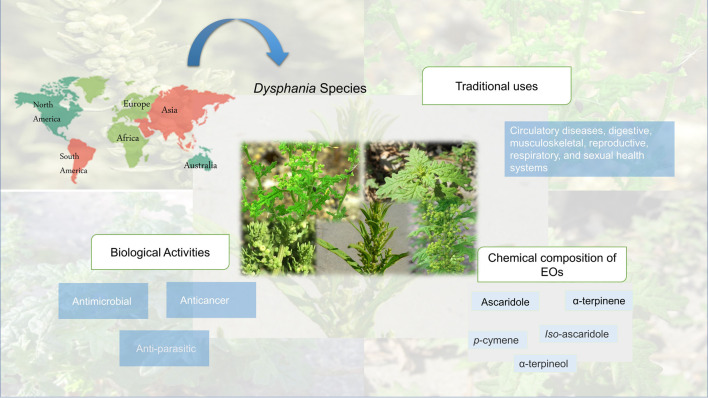

## Introduction

Since antiquity, natural molecules from various sources have been used to cure human ailments (Hassan et al., 2012; Murray et al., 2013; Kola-Mustapha et al., 2020). Among the most significant biomolecule sources are the derivatives of aromatic medicinal plants. As a result, multiple studies have shown that bioactive chemicals from plants have a promising benefic health effect. Among these is the Amaranthaceae family, which is distinguished by the diversity of produced secondary metabolites. This family contains over 175 genera and 2,000 herb species (Mroczek, 2015). The genus *Dysphania* is known for its many pharmacological and preclinical properties. Hence, it is commonly available worldwide and includes more than 47 species (Kim et al., 2019).

*D. ambrosioides* is known as one of the most important species of the *Dysphania* genus, used in the food, cosmetic, and pharmaceutical industries, and also used in traditional medicine to treat several foods (Hallala et al., 2010; Kasali et al., 2021), followed by *Dysphania botrys* (syn. *Chenopodium botrys*)*,* which represents the second species most studied in the literature (Morteza-Semnani, 2015) *Dysphania multifida, Dysphania schraderiana*, *and Dysphania pumilio* are still less studied. The chemical composition of *Dysphania* essential oils (EOs) depends on different environmental factors (Barra, 2009). However, the composition of all the EO examined was different, with a significant quantity of monoterpene compounds (Brahim et al., 2015; Zefzoufi et al., 2020). *Dysphania* EO is also antibacterial (Kandsi et al., 2022), antifungal (Chekem et al., 2010), anti-oxidant (Villalobos-Delgado et al., 2020), and antiviral (Arena et al., 2018).

To the best of our knowledge, there are no reports in the literature that provide a comprehensive analysis of *Dysphania* species. In an effort to better understand its current research status and justify the further exploration and comprehensive application of this genus, we review the botanical, ethnopharmacological, chemical composition, and pharmacological activities of *Dysphania* spp., in addition to its distribution and its possible mechanisms of action and toxicology.

## Methodology

**FIGURE 1:**
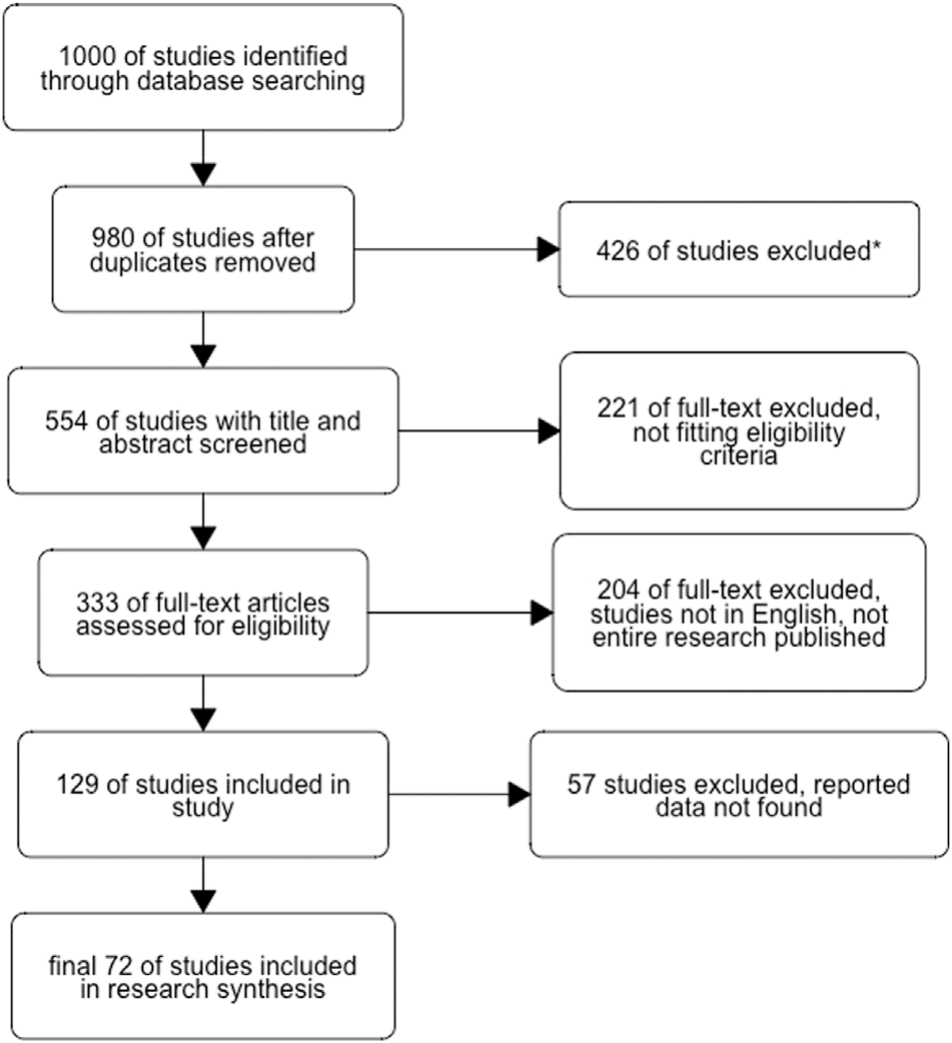
Flowchart of the study design and the bibliographic sources selection process. The search protocol using keywords selection (EO of *Dysphania* chemical composition, bioactivity, and toxicity) resulted in 1,000 publications; 20 duplicates were removed; 426 studies were excluded due to the presence of abstract, citations, and thesis; 221 full-text excluded not fitting eligibility criteria with the topic field out of our study aim, 204 were excluded when not the entire research published, studies not in English, and 57 studies were excluded because the data reported have been not founded. *The figure was done using R metagear package.*

We searched for published articles and grey literature (e.g., unpublished studies, theses, reports, and conference abstracts) that fit these two search criteria: 1. Original research articles with hypothesis tested in the laboratory (e.g., *in vitro*, *in vivo*, preclinical studies) assessing the essential oils’ biological activities and toxicology of the *Dysphania* genus, and 2. studies published in English with full *pdf files available. There were no restrictions on the publication dates of the selected papers, which included both contemporary and older works, to collect extensive data for the review. Using Science Direct, PubMed, ResearchGate, Google Scholar, and Web of Science (WOS: 22 July 2022 with University Hassan II of Casablanca institutional subscription), we found 1,000 publications (Figure 1) with this keyword search: ((*Dysphania*) AND (ethnopharmacology OR pharmacology*) (activity* OR bio* activity) AND (toxicology*)).

This paper has chosen, evaluated, and discussed a few selected publications. After duplicate removal, excluded studies that were not in our specific aim, and excluded reports resulted in 333 studies.

We established the requirements for the studies’ selection; articles with extensive studies on the *Dysphania* essential oils (EOs) composition, therapeutic uses, biological, and pharmacological activities, as well as toxicity, were eligible for inclusion. The exclusion criteria were as follows: if the topic field is not our aim, not the entire research has been published, and studies that were not published in English. We found the essential data/results/papers on the subject, which resulted in 129 publications included in the screening, from which 57 have only an abstract or the title, with no available *pdf files. We conducted the selection procedure for the most relevant articles for this research based on this selected article.

## *Dysphania* genus

Currently, *Dysphania* genus belongs to the new classification, which aggregates the Chenopodiaceae-Amaranthaceae in a single-family known as Amaranthaceae according to the APG III system (Group, 2009), this genus comprises more than 47 species. The representatives of the genus are mainly ruderal and weed plants, more common in the tropics, subtropics, and warm-temperate zones (Judd and Ferguson, 1999; Sukhorukov et al., 2016). Five species have been reported in the literature; *D. ambrosioides*, *D. botrys*, *D. multifida*, *D. schraderiana*, *and D. pumilio* (Mosyakin and Clemants, 2002; The Plant List, 2020). The *Dysphania* species are known to generate glandular white hairs and yellow or orange subsessile glands. These glands contain essential oils that give off a distinctive aromatic odor that frequently remains in herbarium specimens for years (Uotila et al., 2021).

## Distribution

*Dysphania* Spp., are pervasively distributed throughout both temperate and tropical parts of the world. This genus became more widespread due to its ability to adapt to a variety of ecological conditions. There are two majors domesticated *Dysphania*, *D. ambrosioides*, and *D. botrys*. These two species have been cultivated over vast areas of the old world (Sukhorukov et al., 2016). Table 1 provides a list of the common *Dysphania* species distribution.

**TABLE 1:** Geographical distribution of some common *Dysphania* Spp.

Continent	Species	Regions
Africa	*D. ambrosioides* (L.) Mosyakin and Clemants	Southern Africa/North Africa
	*D. multifida* (L.) Mosyakin and Clemants	North Africa
	*D. botrys* (L.) Mosyakin and Clemants	Mountainous tropical Africa
	*D. schraderiana* (Schult.) Mosyakin and Clemants	East and Central Africa
	D. pumilio (R.Br.) Mosyakin and Clemants	Congo
	*D. procera* (Hochst. ex Moq.) Mosyakin and Clemants	East and Central Africa
	*D. congolana* (Hauman) Mosyakin and Clemants	East and Central Africa
	*D. pseudomultiflora* (Murr) Verloove and Lambinon	Southern Africa
Asia	*D. ambrosioides* (L.) Mosyakin and Clemants	India/China
	*D. multifida* (L.) Mosyakin and Clemants	India
	*D. botrys* (L.) Mosyakin and Clemants	China/India/pakistan
	*D. schraderiana* (Schult.) Mosyakin and Clemants	Southeast Asia
	D. pumilio (R.Br.) Mosyakin and Clemants	Southeast Asia/India
	*D. bhutanica* Sukhorukov	Southeast Asia
	D. nepalensis (Link ex Colla) Mosyakin and Clemants	Nepal
	*D. kitiae* Uotila	China
	*D. neglecta* Sukhorukov	Southeast Asia
	*D. geoffreyi* Sukhor	Himalayas and Tibet
	*D. himalaica* Uotila	Himalayas and Tibet
Australia	*D. congestiflora* S.J.Dillon and A.S.Markey	Western Australia
	D sphaerosperma Paul G.Wilson	Western Australia
	D. plantaginella F.Muell.	South Australia
	*D. carinata* (R.Br.) Mosyakin and Clemants	Eastern Australia
	*D. cristata* (F.Muell.) Mosyakin and Clemants	Australia
	*D. glandulosa* Paul G.Wilson	Western Australia
	*D. glomulifera* (Nees) Paul G.Wilson	Australia
	*D. kalpari* Paul G.Wilson	Central Australia
	*D. littoralis* R.Br	Eastern Australia
	*D. melanocarpa* (J.M.Black) Mosyakin and Clemants	Australia
	*D. platycarpa* Paul G.Wilson	Central Australia
	*D. rhadinostachya* (F.Muell.) A.J.Scott	Australia
	*D. pumilio* (R.Br.) Mosyakin and Clemants	Australia
	*D. saxatilis* (Paul G.Wilson) Mosyakin and Clemants	Western Australia
	*D. simulans* F.Muell. and Tate	Central Australia
	*D. sphaerosperma* Paul G.Wilson	Central and Western Australia
	*D. truncata* (Paul G.Wilson) Mosyakin and Clemants	Central Australia
	*D. valida* Paul G.Wilson	Eastern Australia
Europe	*D. ambrosioides* (L.) Mosyakin and Clemants	Italy/France
	*D. multifida* (L.) Mosyakin and Clemants	Bulgaria
	*D. botrys* (L.) Mosyakin and Clemants	Bulgaria/France
	*D. schraderiana* (Schult.) Mosyakin and Clemants	Poland
	D. pumilio (R.Br.) Mosyakin and Clemants	Italy/Romania
America	*D. ambrosioides* (L.) Mosyakin and Clemants	South America
	*D. multifida* (L.) Mosyakin and Clemants	South America
	*D. botrys* (L.) Mosyakin and Clemants	North America
	*D. cristata* (F.Muell.) Mosyakin and Clemants	North America
	*D. anthelmintica* (L.) Mosyakin and Clemants	Southern U.S.A., Mexico, and West Indies
	*D. atriplicifolia* (Spreng.) G.Kadereit, Sukhor. and Uotila	Mexico, and the U.S.A.
	*D. chilensis* (Schrad.) Mosyakin and Clemants	South America
	*D. graveolens* (Willd.) Mosyakin and Clemants	Mexico and the southern U.S.A

## Botanical description

*D. ambrosioides* (L.) Mosyakin and Clemants, is the most well-known species from this genus, represents an annual or short-lived perennial herbaceous plant, with a strong odor, which reaches up to 1 m high, with erect stems, very branched, alternate leaves elongated with acute apex, edges serrated, hairy, of different sizes sessile; racemose inflorescence presenting small white flowers with 3–5 free or united sepals and 3 to 5 free or rarely adnate stamens, compressed spherical ovary and many black seeds (with a length less than 0.08 mm) (Sá et al., 2016; Paniagua-Zambrana et al., 2020).

*D. botrys* (L.) Mosyakin and Clemants, is a naturally growing wild plant, traditionally used by rural and endemic inhabitants, has a characteristic odor due to the presence of sesquiterpenes and monoterpenes, and is an annual plant of 20–50 cm, stem erect, angular, branching often from the base, with erect-spreading branches, lower leaves long petiolate, pinnately lobed, racemose inflorescence of a yellowish green (Khan and January 2019).

*D. multifida* (L.) Mosyakin and Clemants, commonly known as “paico”, is an aromatic plant widely used for medicinal purposes, perennial plant of 30–80 cm, pubescent, with a penetrating and pleasant smell, stems numerous, very branchy, leaves small, puberulous-glandulosa, shortly petiolate, with lanceolate or linear lobes, greenish (Yossen et al., 2019).

*D. schraderiana* (Schult.) Mosyakin and Clemants, this plant is used in a variety of applications such as medicine. Pubescent annual (height: 20–60 cm), oblong leaves (long: 2–6 cm, wide: 1.5–3.5 cm), attenuated base, obtuse to acuminate apex, pinnately lobed margins, glabrescent petiole (2–10 mm long). Flowers with 5 oval sepals (long: 1 mm), and 5 stamens, are grouped in inflorescence (Łuczaj et al., 2022).

**FIGURE 2:**
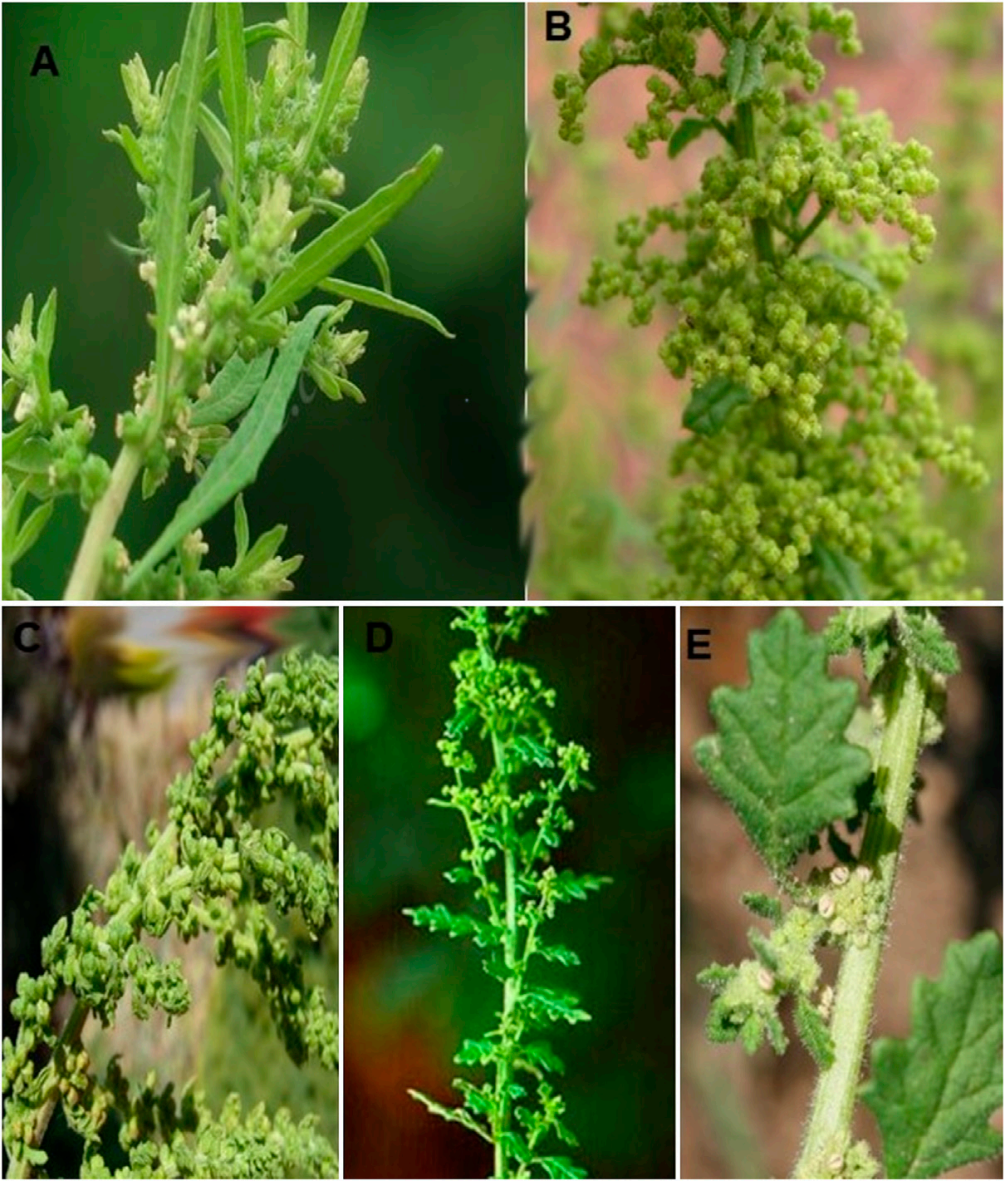
*Dysphania* species. **(A)** *D. ambrosioides*, **(B)** *D. botrys*, **(C)** *D. mutifida*, **(D)** *D. schraderiana*, **(E)** *D. pumilio*.

*D. pumilio* (R.Br.) Mosyakin and Clemants, is one of the popular invasive species, pubescent annual (height: 5–45 cm). Leaves ovate to elliptical (length: 0.5–2.5 cm, width: 0.5–1.5 cm), wedge-shaped base, obtuse apex, entire margins, glabrescent petiole (3–15 mm) (see Figure 2). Flowers with 5 elliptical to oblong sepals (long: 1 mm), and 1 stamen sometimes absent, grouped in inflorescence (diameter: 2–3 mm) assembled in axillary cymes (length: 3–7 cm) (Bogosavljević and Zlatković, 2017).

## Ethnopharmacology

Since ancient times, *Dysphania* species have been used around the world to cure various ailments (Table 2), specifically circulatory diseases, digestive, musculoskeletal, reproductive, respiratory, and sexual health systems (Bussmann et al., 2018). Aside from being utilized as an herbal remedy, some plants of this genus may be consumed due to their nutritional components. The leaves, fruits, and flowers can also be made into different food products. For example, they are used as spices in different countries (Barragán and Carpio, 2009; Barros et al., 2013). Traditional uses of *Dyphania* Spp., are represented in Table 2.

**TABLE 2:** Traditional uses of *Dysphania Spp*.

Species	Ethnomedical uses	Used parts	Method of preparation	References
*D. ambrosioides*	Gastrointestinal disorders, typhoid, dysentery, galactogen, oral abscesses, ulcers, purulent wounds, and diabetes.	W.P	Infusion	(Hallala et al., 2010; Brahim et al., 2015).
			Decoction	
			Poultice	
*D. multifida*	Digestive and antiparasitic	L	Infusion	Yossen et al. (2019).
			Condiment	
*D. botrys*	Asthma, cough, wounds, fever, pain, liver, respiratory, urinary, and gastric complaints, as an antiseptic and for wound healing	S	Infusion	Khan and Jan, (2019).
			Decoction	
*D. schraderiana*	Reducing wheezing, inflammation, cramping, and migraines	L	Infusion	Łuczaj et al. (2022).
*D. pumilio*	Nr*	Nr*	Nr*	—

Legend: *Nr, not reported.

## Chemical composition

Several studies have revealed that *Dysphania* is an important genus with various compounds, especially essential oils. The most prevalent were monoterpenes, and sesquiterpenes (Kokanova-Nedialkova et al., 2009; Barros et al., 2013). Currently, approximately 45 terpenoid compounds have been reported and isolated from the fruits, seeds, leaves, and flowers of *Dysphania* species EO. The main chemical compounds occurring in the essential oils obtained from the *Dysphania* genus are represented in Table 3.

**TABLE 3:** Chemical composition of *Dysphania* Spp., plant essential oils.

Species	Chemical compounds	References
*D. ambrosioides*	α-terpinene: 23.77%	Brahim et al. (2015)
	Ascaridole: 14.48% *p*-cymene: 12.22%	
*D. multifida*	α-terpinene: 18.5%	Yossen et al. (2019)
	Ascaridole: 61.1% *p*-cymene: 12.7%	
*D. botrys*	α-terpineol: 52.8%	Morteza-Semnani, (2015)
	*Iso*-ascaridole: 7% *p*-cymene: 19%	
*D. schraderiana*	Nr*	—
*D. pumilio*	Nr*	—

Legend: *Nr, not reported.

**FIGURE 3:**
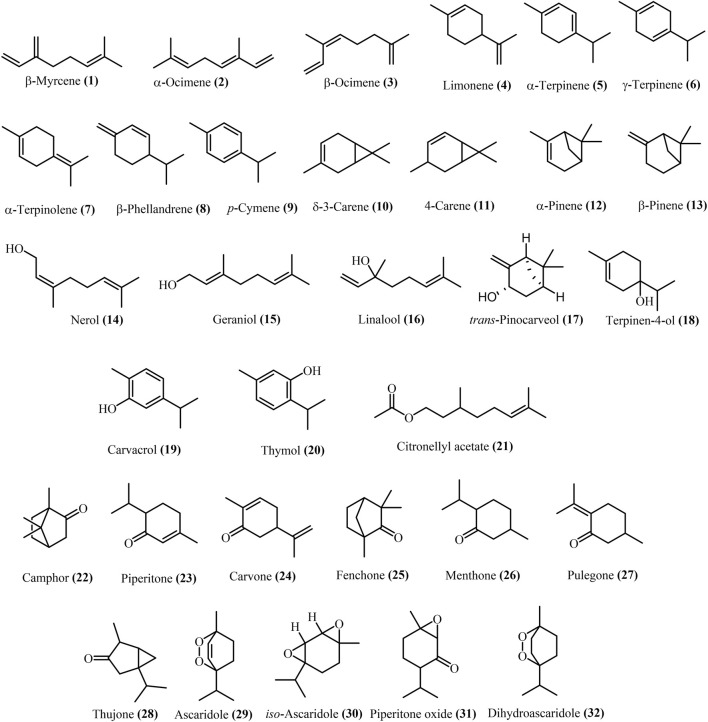
Monoterpenes of *Dysphania spp* essential oils.

Approximately 44 papers covered the *Dysphania* EO assessment. The majority of the paper identified the components of *D. ambrosioides* EO are oxygenated monoterpenes. In several studies (Gupta et al., 2002; Boutkhil et al., 2009; Brahim et al., 2015; Bisht and Kumar, 2019), α-terpinene **(5)** was quantified as the main constituent in *D. ambrosioides* EO, while ascaridole **(29)** was reported as the most abundant components in *D. multifida* EO (Yossen et al., 2019). Less frequently, δ-3-carene **(10)**, limonene **(4)**, thymol **(20)**, carvacrol **(19)**, γ-terpinene **(6)**, α-terpinolene **(7)**, piperitone oxide **(31)**, geraniol **(15)**, α-pinene **(12)**, β-pinene **(26)**, iso-ascaridole **(20)**, β-myrcene **(1)**, α-ocimene **(2)**, β-ocimene **(3)**, citronellyl acetate **(21)**, β-phellandrene **(8)**, dihydroascaridole **(32)**, *trans*-pinocarveol **(17)**, carvone **(24)**, piperitone **(23)** were reported in *D. multifida* and *D. ambrosioides* EO (Arena et al., 2018), while *p*-cymene **(9)**, and 4-carene **(11)** were reported as main components of *D. ambrosioides* EO in another study (Zefzoufi et al., 2020). Other compounds, camphor **(22)**, δ-3-carene **(23)**, fenchone **(25)**, linalool **(16)**, menthone **(26)**, nerol **(14)**, β-pinene **(13)**, pulegone **(27)**, terpineol-4-ol **(18)**, thujone **(28)**, and *iso*-ascaridole **(30)** are represented in *D. botrys* EO. The structures of monoterpenes from **1** to **32** are shown in Figure 3.

**FIGURE 4:**
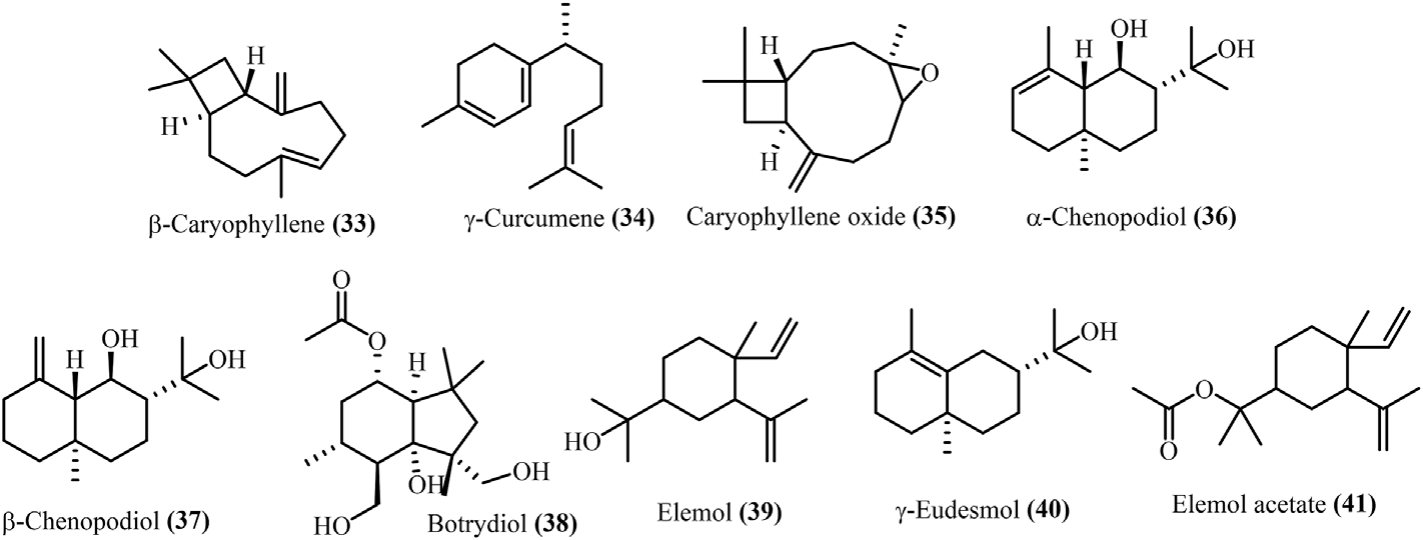
The Structure of the main sesquiterpenes in *Dysphania spp* essential oils.

Major sesquiterpenes in *D. ambrosioides* included β-caryophyllene **(33)**, *γ*-curcumene **(34)*,*** and caryophyllene oxide **(35)** (Kokanova-Nedialkova et al., 2009). While, *D. botrys* included elemol **(39)**, elemol acetate **(41)**, *α*-chenopodiol **(36)**, *β*-chenopodiol **(37)**, botrydiol **(38)**, and eudesmol **(40)** are shown in Figure 4. These main sesquiterpenes were identical across different *Dysphania* populations based on GC-MS data, although relative quantity varied (Pino et al., 2003; Singh et al., 2008).

In addition, many intrinsic and extrinsic factors, such as environmental factors, affect the *D. ambrosioides* essential oils yield and constituents. Plants may be stressed due to high or low salinity, causing a change in the content of EO (Verma and Shukla, 2015). According to several authors (de Carvalho et al., 2018b), the amount of four main volatile constituents (α-terpinene, *p-*cymene, *E*-ascaridole, and *Z*-ascaridole) is affected by salt concentrations. Salts are essential to plant growth and metabolism. High concentrations may be toxic (Mosa et al., 2017). The blue LED was also shown to block the production of ascaridole **(29)** (53.21%), whereas fluorescent light increased the conversion of α-terpinene **(5)** to ascaridole **(29)** (de Carvalho et al., 2020). In general, these results agree with the observation that many enzymes of the secondary pathways are light-dependent (Yabuta et al., 2007; Alvarenga et al., 2015). Another study (Yousefi et al., 2011) showed that the development stages of *D. botrys* are affected by heavy metals. Treatments without CaCl_2_ and MgSO_4_ had an antagonistic connection with *p-*cymene **(9)**, and treatments with MgSO_4_ at 1,480 mg L^−1^ gave higher levels of ascaridole **(19)**. KH_2_PO_4_ at a concentration of 680 mg L^−1^ caused an excess of ascaridole **(29)** to be found in the treatment. α-terpinene **(5)** represents a significant amount in treatment by CaCl_2_ at a concentration of 880 mg L^−1^ (de Carvalho et al., 2018a) ascaridole **(29)** content in the leaves increased when quail manure was used, whereas it increased in the inflorescences when chicken manure was used (Bibiano et al., 2019). However, the greatest *α*-terpinene **(5)** content was reported without using chitosan. According to the biosynthetic pathway, chitosan and salicylic acid favoured the conversion of α-terpinene **(5)** to ascaridole **(29)** (Dembitsky et al., 2008; de Carvalho et al., 2020). This paper mainly focused on C_10_ monoterpenes, and C_15_ sesquiterpenes for their importance. All these terpenoids are derived from two distinct biochemical pathways; the (MEP) 2C-methyl-D-erythritol-4-phosphate pathway, which is active in the plastids, begins from pyruvate and glyceraldehyde-3-phosphate, whereas the (MVA) mevalonic acid pathway active in the cytosol and starts from acetyl CoA (Bergman et al., 2019).

## Health benefits

### Antimicrobial effects

Bacteria have evolved several mechanisms to withstand antibiotic action. Several investigations have indicated that *D. ambrosioides L.* has inhibitory action against a wide spectrum of pathogenic bacteria*.* Brahim et al., 2015 (Brahim et al., 2015) reported that EO isolated from *D. ambrosioides* are more active against *Bacillus cereus* and *Micrococcus luteus* than *Klebsiella pneumoniae* and *Pseudomonas aeruginosa* with zones of inhibition ranging from 15.33 to 21.5 mm and from 7.17 to 19.17 mm, for Gram-positive and Gram-negative bacteria, respectively. The cell envelope structure explains this, since Gram-negative bacteria have an additional membrane, limiting hydrophobic compound diffusion. *D. ambrosioides* EO has also been shown to have antibacterial activity against *Helicobacter pylori* (Ye et al., 2015), also, against *Escherichia coli, staphylococcus aureus,* and *Enterococcus faecali*s (Kandsi et al., 2022) with ZI ranging from 9 to 24 mm. *D. botrys* EO also showed strong antimicrobial activity against a variety of bacteria (*Staphylococcus aureus*, *Bacillus cereus, Staphylococcus saprophyticus*, *Klebsiella pneumoniae*, *Staphylococcus epidermidis*, *Streptococcus mutans*, *Listeria monocytogenes,* and *Salmonella typhimurium*) with ZI ranging from (9–22 mm) (Foroughi et al., 2016). Numerous studies evaluated the antifungal activity of *D. ambrosioides* EO against fungal. Brahim et al., 2015 (Brahim et al., 2015) also reported high anticandidal activity, where *Candida albicans* was the most susceptible yeast, having the lowest minimum inhibitory concentration. Likewise, Mokni et al., 2019 (Mokni et al., 2019) observed that *D. ambrosioides* EO exhibited considerable antifungal activity against *Candida albicans*. Similarly, good activity was recorded for *D. botrys* EO on *C. albicans* and showed an inhibitory effect on *Aspergillus* species and *Bacillus subtilis* (Mahboubi et al., 2011), while for *Trichophyton mentagrophytes, Epidermophyton floccosum, Candida albicans, Aspergillus niger,* and *Microsporum canis. D. botrys* EO showed ZI ranging from (14–20 mm) (Tzakou et al., 2007). Available scientific data have shown consistent findings from several authors. The following main points have evolved as a result of this: These plants EO have good antimicrobial activity against a wide range of pathogens, including Gram-negative and Gram-positive bacteria and fungi, this high activity has been linked to the presence of monoterpene hydrocarbons (limonene **(4)**, *p*-cymene **(9)**, and ascaridole **(29)**, thymol **(20)**, carvacrol **(19)**, and α-terpinene **(5)**). All mechanisms described in the literature show that *Dysphania* EO affects the cellular integrity of bacteria, a decrease in respiration, and an alteration in permeability. Few studies have described the antimicrobial activities from other *Dysphania* species (Table 4). The inhibitory effectiveness of *Dysphania* EO against microbial growth is stronger than reference antimicrobials even in experiments with the positive control, hence, EO from this species can be advised as a replacement for conventional antimicrobial agents. It should be noted that most research on the antibacterial properties of *Dysphania* spp. has been conducted *in vitro*, which does not ensure that the results would be the same *in vivo*. Furthermore, the susceptibility testing in the aforementioned research solely employed traditional techniques. However, additional techniques may be modified to determine the antimicrobial susceptibility of EO, including bioautography, flow cytometry, and bioluminescence experiments.

**TABLE 4:** Biological effects of *Dysphania Spp*.

Activity	Species	Plant part	Source	Dosage/Duration	Model	Positive controls	Mechanisms	References
Antibacterial	*D. ambrosioides*	Whole plant	EO	500 μg ml^−1^	*in vitro*	Norfloxacin Tetracycline Lansoprazole Metronidazole Clarithromycin	Alteration of bacterial cellular integrity and permeability;	de Morais Oliveira-Tintino, Tintino, Limaverde, Figueredo, Campina, da Cunha, da Costa, Pereira, Lima, and de Matos (2018)
		Leaves		49.32 mg kg^1^	*in vivo*			
		Aerial parts		2 weeks			Inhibition of respiration.	Limaverde et al. (2017)
	*D. botrys*	Aerial parts	EO	98.6 μg ml^−1^	*in vitro*	Kanamycin Cephalexin	Reduction of efflux pump in *Staphylococcus aureus*	Foroughi et al. (2016)
Antifungal	*D. ambrosioides*	Leaves	EO	0.25–2 mg ml^−1^	*in vitro*	Ciprofloxacin	Increase the membrane permeability	(Brahim et al., 2015; P. Singh and Pandey 2021; Prasad et al., 2010)
		Whole plant		0.1%, 1% and 10%	*in vivo*			
		Aerial parts		7–21 days				
	*D. botrys*	Aerial parts	EO	4 μL ml^−1^	*in vitro*	Vancomycin		
						Gentamicin		
						Amphotericin B		
Antioxidant	*D. ambrosioides*	Leaves	EO	500 μg ml^−1^	*in vitro*	Quercetin	Upregulation or protection of antioxidant defenses, scavenging of reactive oxygen species, and suppressing their formation through both enzyme inhibition and chelation of trace elements involved in a free radical generation	(Bezerra et al., 2019; Kandsi et al., 2022)
						BHT		
Antiviral	*D. ambrosioides*	Leaves	EO	21.75 μg ml^−1^	*in vitro*	Human Coxsackie virus-B	NR*	Mokni et al. (2019)
					*in silico*			
Antileishmanial	*D. ambrosioides*	Leaves	EO	30 mg kg^−1^	*in vivo*	Chloroquine	Inhibition of NADH^−^Reduction of succinate-dependent cytochrome C	(Monzote et al., 2014; Machín, Tamargo, Piñón, et al., 2019)
						Benznidazol		
				14 days		Suramine	Generation of oxygen radicals, mitochondrial dysfunction, and a modification of redox indexes	
						Miltefosine		
Amoebicidal	*D. ambrosioides*	Leaves	EO	0.75 mg ml^−1^	*in vitro*	Metronidazole	Endoperoxide that it can deliver reactive oxygen species and damage the trophozoites in a similar way that oxygen peroxide induces toxicity to amoeba free radical-triggered DNA or protein alterations	Ávila-Blanco et al. (2014)
				8 mg kg-^1^				
				80 mg kg^−1^	*in vivo*			
				7 days				
Insecticidal	*D. ambrosioides*	Whole plant	EO	8.80 μg L^−1^	*in vitro*	Acetone	Inhibition of GSTs and CarE activity;	Wei et al. (2015)
							Disrupted the activities of some endogenous protective enzymes (SOD, POD, CAT);	
		Aerial parts		2.437 mg L^−1^	*in vivo*		Interfere with the neuromodulator octopamine;	
				24h/48 h			Modulate GABA-gated chloride channels.	
Nematicidal	*D. ambrosioides*	Fruits	EO	500 μg ml^−1^	*in vitro*	Carbofuran	Reduction in hatching of a nematode	(A.F. Barros et al., 2019)
		Seeds		7 days	*in vivo*			
Anticancer	*D. ambrosioides*	Whole plant	EO	50 μg ml^−1^	*in vitro*	DMSO	Affects antioxidant system of cancer cells	Ya-Nan et al. (2015)
				125 μg ml^−1^				
				31.25 μg ml^−1^	*in vivo*			
				24 h				
Wound healing	*D. botrys*	Leaves	EO	1 ml	*in vivo*	Tetracycline	NR*	Sayyedrostami et al. (2018)
				10 days				
Molluscicidal	*D. ambrosioides*	Leaves	EO	2.40 and 8.75 ppm	*in vivo*	NR*	Alteration in mitochondrial membrane potential, causing oxidative phosphorylation breakdown and modification of redox indexes	Ignacchiti et al. (2022)
	*D. ambrosioides*	Leaves	EO	49.32 mg kg^1^	*in vivo*	Lansoprazole	NR*	Ye et al. (2015)
						Metronidazole,		
				2 weeks		Clarithromycin		
Relaxant	*D. ambrosioides*	Leaves	EO	1,000 μg/ml	*in vivo*	Nifedipine	Block the KCl-induced contractile response	Pereira-de-Morais et al. (2020)
				5–15 min				

Legend: *Nr, Not reported.

### Antiviral effects

One of the common viruses is enteroviruses, specifically the Coxsackie B4 virus (CVB4) enteroviruses that belong to the *Picornaviridae* family, which is associated with serious illnesses, including myocarditis and meningoencephalitis (Sin et al., 2015). In this context, the EO obtained from *D. ambrosioides* L., growing wildly in Tunisia, demonstrated a significant antiviral effect against the CV-B4 virus. This activity could be attributed to ascaridole **(29)** (Mokni et al., 2019). However, more research *in vitro* and *in vivo* is needed to evaluate the antiviral activity of EO and their active compounds isolated from all *Dysphania* spp.

### Anti-leishmanial effects

The hunt for effective therapeutics to treat Leishmaniasis has become an urgent requirement due to the absence of effective medicines and the limits of present treatments (Machín et al., 2019). The anti-leishmanial activity of *D. ambrosioides* was demonstrated by Monzote et al., 2014 and 2018 (Monzote et al., 2014; Monzote et al., 2018) against amastigotes and promastigotes of *Leishmania amazonensis.* Results show a more significant inhibitory effect of ascaridole **(29)**. This effect is by reducing succinate-dependent cytochrome C due to the inhibition of NADH. To more understand the effects of *D. ambrosioides* EO and resolve the stability and solubility problems of EO, some studies (Machín et al., 2019) aim to explore the encapsulation of *D. ambrosioides* L. EO in nanocochleates (lipid-based delivery system) and investigated *in vitro* and *in vivo* against *L. amazonensis*. The results showed that *D. ambrosioides* L. EO-nanocochleates (NC) did not affect the EOs’ *in vitro* inhibitory efficacy. The formulation caused no mortality or weight loss higher than 10% in the animal model (Table 4). Mice treated with *D. ambrosioides* EO-NC had more extensive lesions than those treated with EO. This activity may be related to the presence of some terpenoid compounds. Hence, the results showing a potent anti-leishmanial activity from *in vitro* and *in vivo* indicating a safe application as drug.

### Antioxidant effects

Several studies showed that *D. ambrosioides* L. EO had an essential antioxidant activity. Santiago et al., 2016 (Santiago et al., 2016) reported this activity by different methods DPPH and β-carotene/linoleic acid, showed higher activity of EO in the β-carotene/linoleic acid test. Also, Brahim et al., 2015 (Brahim et al., 2015) demonstrated that *D. ambrosioides* L. EO exhibits free radical scavenging activity by using the DPPH test. Indeed, they found the highest antioxidant capacity by inhibiting lipid peroxidation *via* a β-Carotene/linoleic acid bleaching test. Also, Brahim et al., 2015 (Brahim et al., 2015) marked the high activity by reducing potency (Table 4). The potent antioxidant activity of *Dysphania* EO can be due to its high content of α-terpinene **(5)**, which is characterized by its powerful antioxidant capacity that is probably attributed to the presence of strongly activated methylene groups (Table 4). However, the *in vitro* assays for measuring antioxidant activity have little pharmacological significance and only partially validate the biological impact, more studies *in vivo* about oxidative stress are needed.

### Anticancer effects

Some studies demonstrate the cytotoxic activity of *D. ambrosioides* L. EO against tumours; the authors have demonstrated that the EO reduced cell growth and were cytotoxic to human breast cancer cell lines MCF-7 in a dose and time-dependent manner, *via* an apoptosis-related mechanism. *D. botrys* EO showed maximum growth inhibitions against the A549 cell line and inhibited the growth of the MCF-7 cell line (Table 4) (Shameem et al., 2019). The reported research has shown the precise anti-tumor mechanisms of *D. ambrosioides* EO, which are related to apoptosis induction (Table 4). Therefore, these results could offer an actual overview on the effects of *Dysphania* EO on tumoural cells. However, in these investigations, the cytotoxic effects of *Dysphania* EO were only assessed in tumour cell lines. There have not been any human clinical trials to look at the pharmacokinetics and therapeutic effects of EO and their compounds on cancer patients. Clinical investigations involving humans and animal models should be the main topics of future study. Moreover, further studies to elucidate the antitumoral effect are required.

The benefic effect of *Dysphania* EO (antimicrobial, antiviral, antifungal, antileishmanial, insecticidal, nematocidal, antioxidant, antitumoral, anti-ulcer, and relaxant) are sown in Table 4.

## Toxicology

The centuries-old use of medicinal plants has shown that some of these plants contain potentially dangerous substances (Ndhlala et al., 2013). *D. ambrosioides* L. is one of the plants described as having a toxicological risk, specially indicated for essential oils (GUYTON, 1946).

**FIGURE 5:**
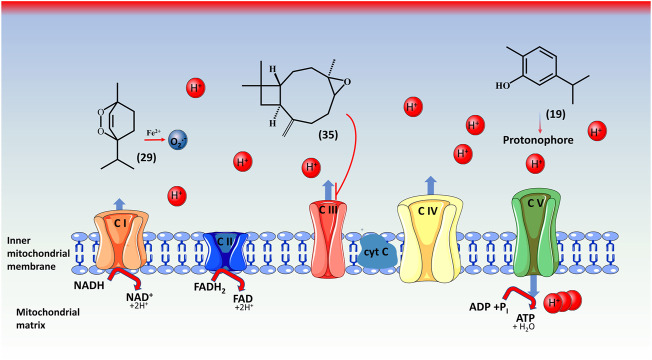
Toxicity mechanisms of ascaridole **(29)**, carvacrol **(19)**, and caryophyllene oxide **(35)** in mitochondria. Both oxidative stress and mitochondrial dysfunction are employed in the mechanism of toxicity by the *Dysphani*a’s EO. The EO have inhibitory effects on mitochondria’s ETC (electron transport chain) complex I-III. Caryophyllene oxide **(35)** carries out inhibition on complex III (CIII). Ascardiole **(29)** following activation by iron (Fe^2+^) threatens mitochondrial uncoupling and triggers superoxide radical formation (O_2_^.-^). Carvacrol **(19)** has no direct inhibiting effects, but a synergistic effect with ascaridole. *complex I: NADH ubiquinone oxidoreductase; complex II: succinate ubiquinone oxidoreductase; complex III: ubiquinol cytochrome c oxidoreductase; complex IV: cytochrome C oxidase; complex V: F_1_F_0_ATP synthase. NADH, nicotinamide adenine dinucleotide hydrogen; NAD^+^, nicotinamide adenine dinucleotide; FADH_2_, flavin adenine dinucleotide (hydroquinone form); FAD, flavin adenine dinucleotide; H^+^, protons; H_2_O, water; H, hydrogen; O, oxygen; Fe, iron. *The figure was produced using Servier Medical Art.*

Several species, including *D. botrys*, and *D. ambrosioides* possess compounds that have been demonstrated to interfere with mitochondrial function (Nagle et al., 2011). The toxicity of EO obtained from *Dysphania* can be associated with the presence of some major components, carvacrol **(19)**, caryophyllene oxide **(35)**, and ascaridole **(29)**, which induce suppression of the respiratory function in the mitochondria, or in the complex I of the mitochondrial electron transport chains (Figure 5) (Monzote et al., 2018), this toxic effect emerging on the kidneys, liver, and intestine (Derraji et al., 2014). Nevertheless, in a recent study by Li et al., 2020 (Li et al., 2020), dose-dependent toxicity was demonstrated in mice, providing some support for using the EO in a safe way in traditional medicine. However, their utilization is contraindicated during pregnancy and breastfeeding for infants under three, and adult patients who are distressed or suffer from liver or renal illnesses (Potawale et al., 2008).

The toxicity of ascaridole **(29)** was observed by activation in the presence of iron, which allows it to be more toxic, resulting in carbon-centred radicals, which are very reactive and can initiate lipid peroxidation and reduce respiration. Caryophyllene oxide **(35)** is the principal generator of superoxide radicals and directly influences complex III. Carvacrol **(19)** reacts as a protonophore and does not have a direct physiological effect. All these actions induce a decrease in ATP production and an increase in superoxide radicals.

## Conclusions and further perspectives

The present review offers the first insights of selected literature regarding the chemical composition of *Dysphania* EOs, their pharmacological properties, and their applications in traditional medicine. Plants of this genus have been used since ancient times to treat many diseases, and these properties have been confirmed by numerous pharmacological studies.

Distinctive chemical constituents have been isolated and identified as belonging to different species. Indeed, the literature has shown that the main components of these essential oils are α-terpinene, ascaridole, *iso*-ascaridole, α-terpineol, and *p-*cymene. Overall, these compounds can change due to abiotic and biotic factors that affect essential oil content and yield. Most chemical studies have focused on the EO content of *D. ambrosioides*, *D. multifida*, and *D. botrys*, while further research on the chemical composition of the EO of other species is needed in order to determine their chemical composition. Determining the bioactivity of other volatile compounds from all species of *Dysphania* would be critical for future investigation and its impact on health. Previous research has revealed the extensive medicinal applications of volatile compounds from different botanical parts of *Dysphania* spp. (seeds, fruits, and leaves) in a range of *in vitro* and *in vivo* test models. *Dysphania* spp. EOs have been demonstrated to possess antibacterial, antifungal, antioxidant, anti-cancer, antiviral, antileishmanial, amoebicidal, and anti-inflammatory properties, and lastly, nematocidal, and insecticidal activities at different doses/concentrations. The chemical composition and pharmacological results validate and support some ethnopharmacological uses of *Dysphania* spp. in traditional medicine.

As this review shown, the *Dysphania* genus EOs, rich in secondary metabolites and various biological activities, can constitute an alternative to certain synthetic drugs to bring health benefits to human diseases in the future. However, according to the literature, current knowledge of *Dysphania* species contains several gaps that require further investigation in preclinical and clinical studies.

## Abbreviations

A.P, Aerial parts; CarE, Carboxylesterase; CAT, catalase; DPPH, 2,2-diphenyl-1-picrylhydrazyl; EO, essential oils; F, Fruits; GST, glutathione-S-transferase; IC50, 50% inhibitory concentration; L, Leaves; MIC, Minimal Inhibition Concentration; POD, Peroxidase; S, Seeds; SOD, superoxide dismutase; W.P, Whole plant; ZI, Zones of inhibition.
